# Supplementary material for: First Record of Colletotrichum anthrisci Causing Anthracnose on Avocado Fruits in Chile
Source: Pathogens. 2022 Oct 19;11(10):1204. doi: 10.3390/pathogens11101204 (PMC9611251; doi:10.3390/pathogens11101204)
Supplement: Supplementary file 1 [file pathogens-11-01204-s001.zip › pathogens-1920499-supplementary Figures.pdf]

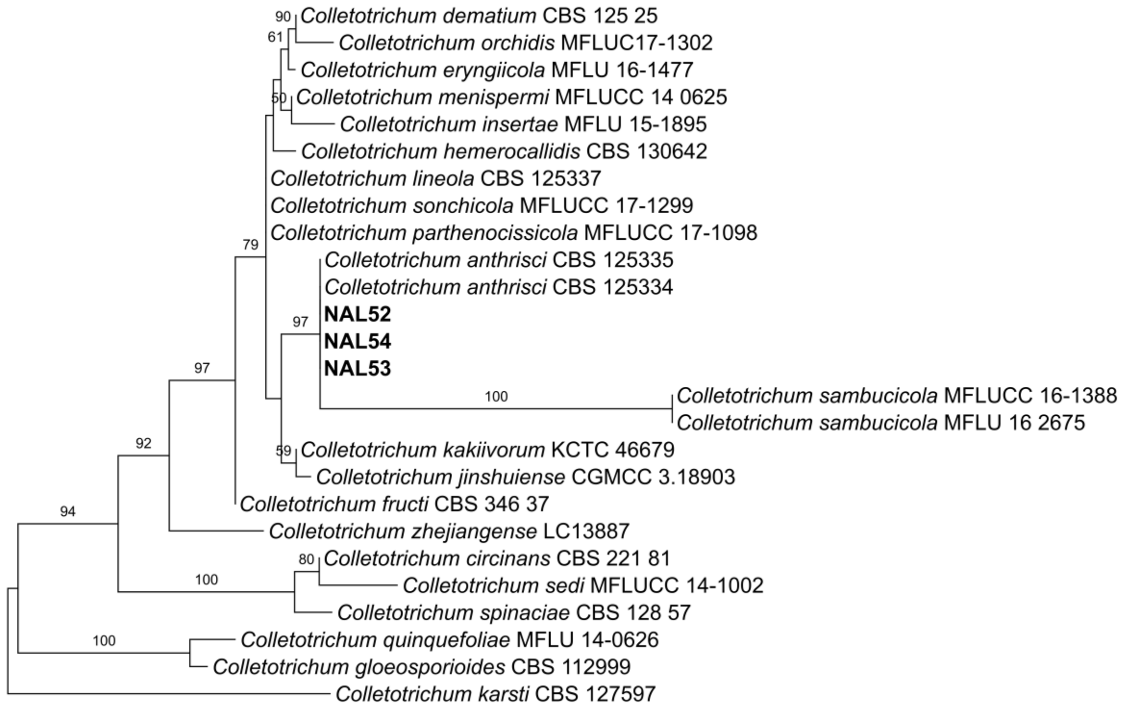

Figure S1. Phylogenetic analysis using *act* sequences.

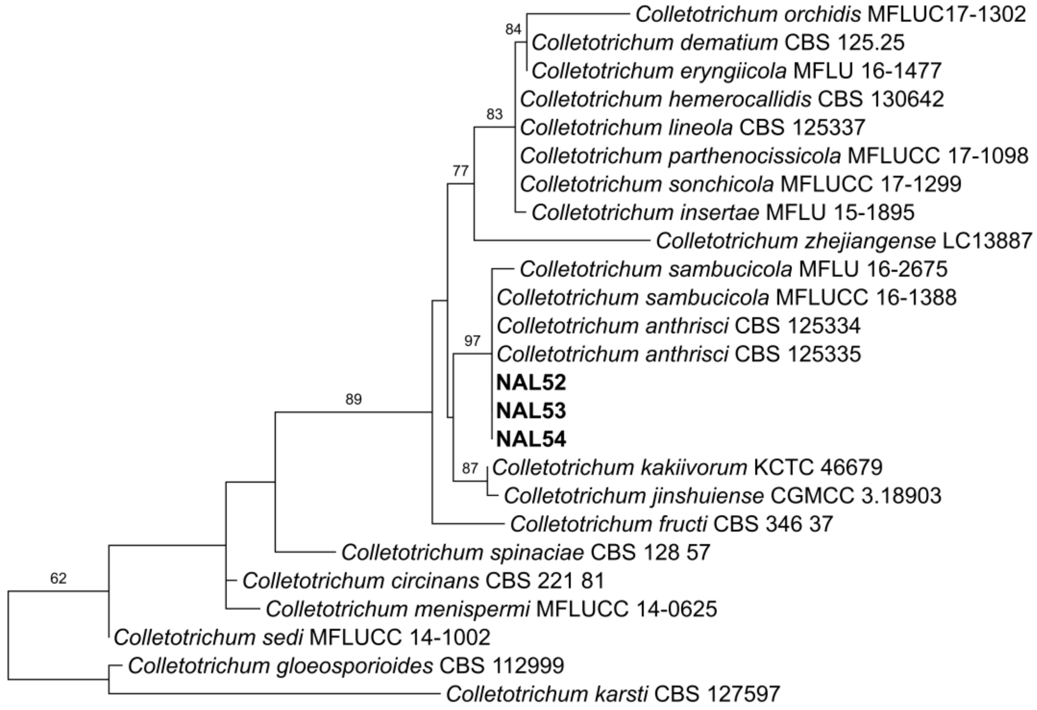

Figure S2. Phylogenetic analysis using *chs-1* sequences.

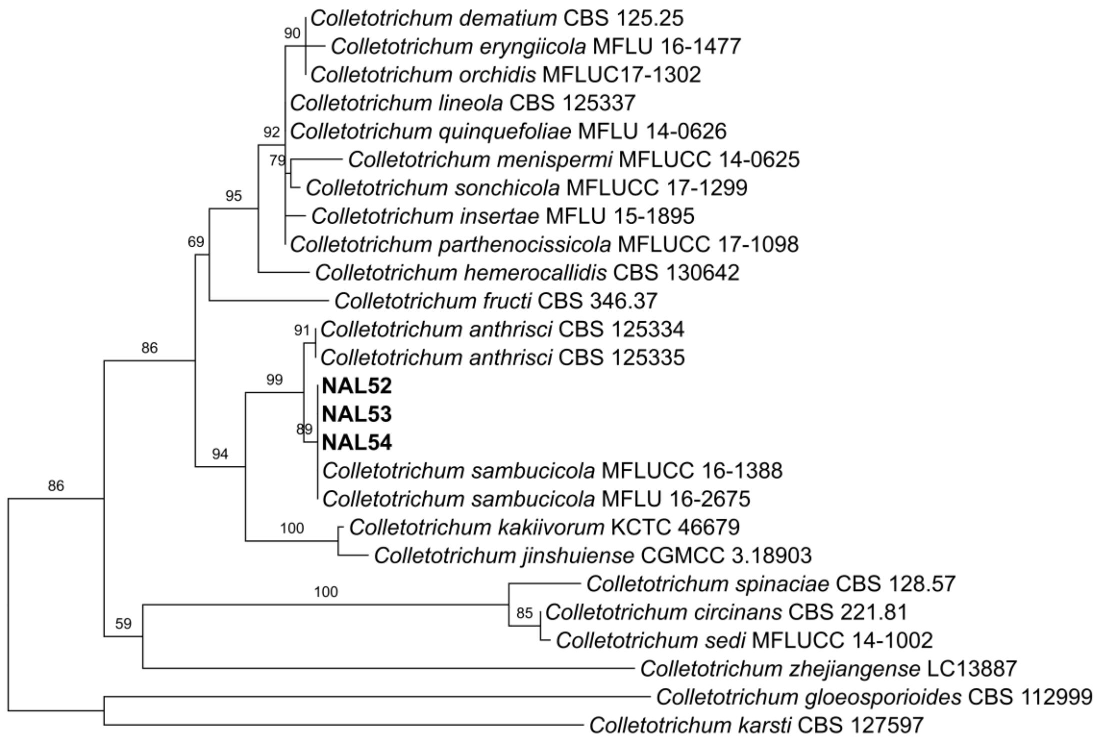

Figure S3. Phylogenetic analysis using *gapdh* sequences.

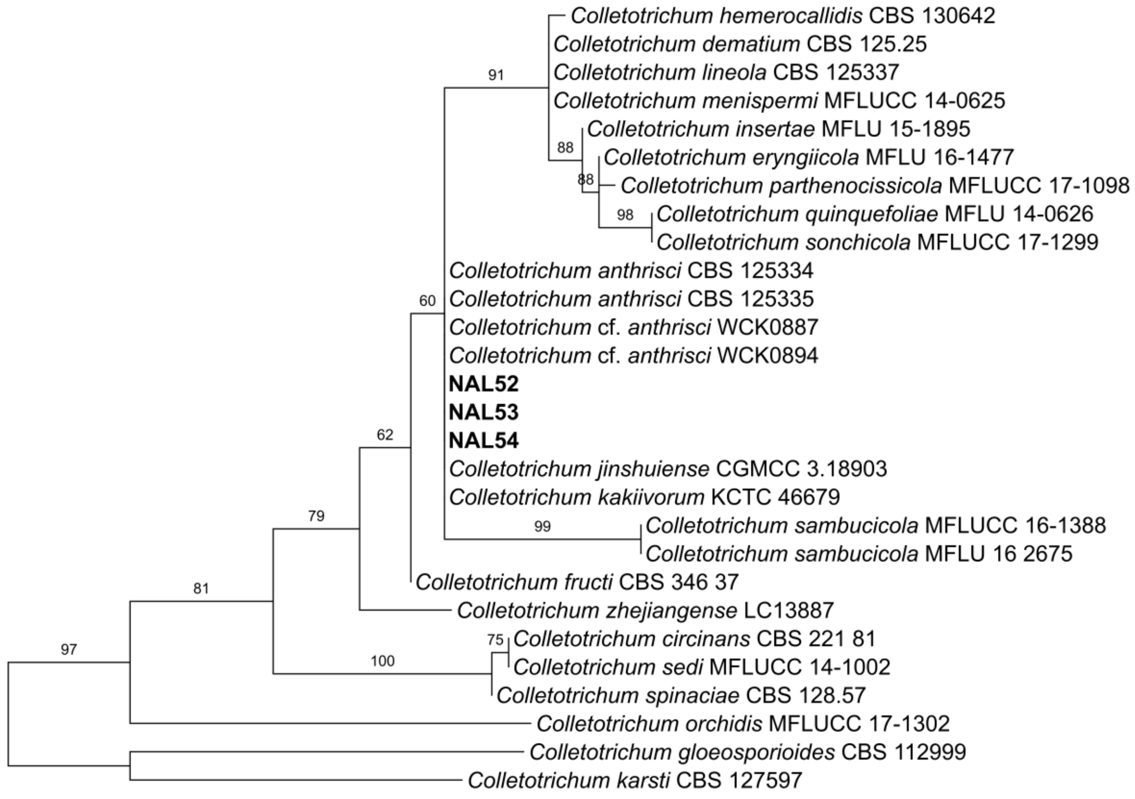

Figure S4. Phylogenetic analysis using ITS sequences.

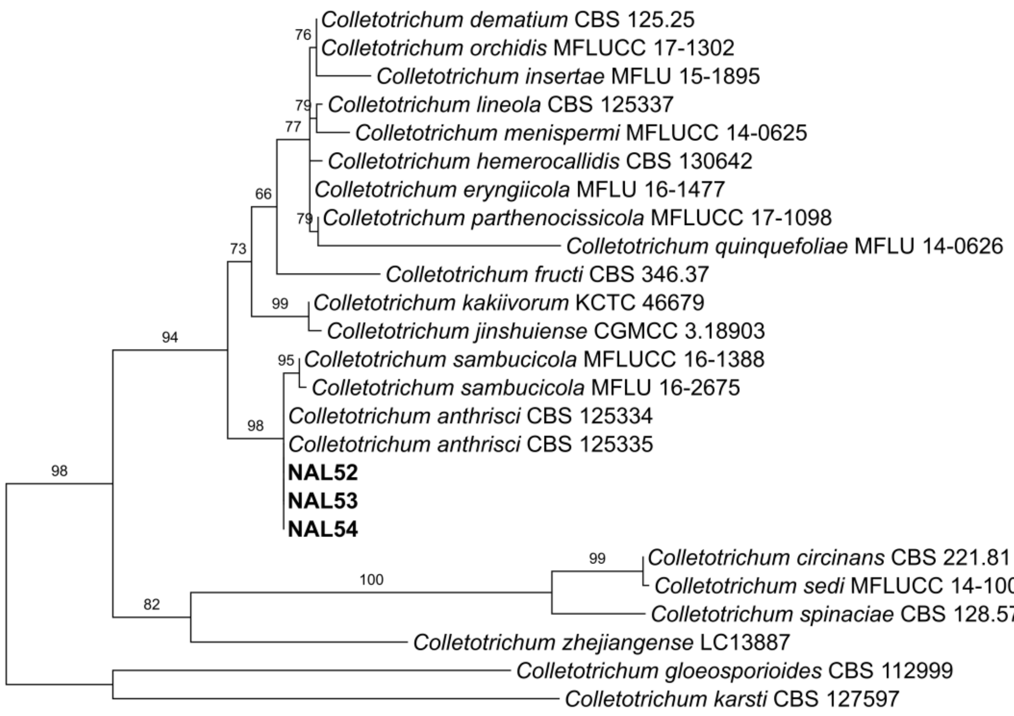

Figure S5. Phylogenetic analysis using *tub2* sequences.

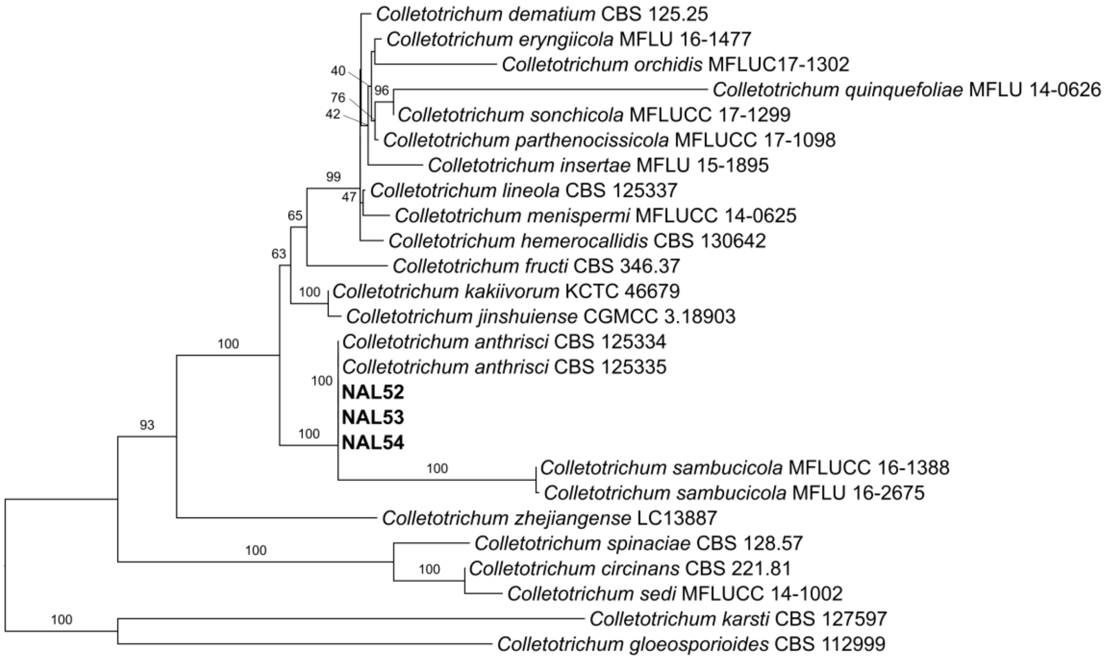

Figure S6. Phylogenetic analysis using four-locus (*act*, *gapdh*, ITS, *tub2*) sequences.
