# Supplementary material for: First Record of Colletotrichum anthrisci Causing Anthracnose on Avocado Fruits in Chile
Source: Pathogens. 2022 Oct 19;11(10):1204. doi: 10.3390/pathogens11101204 (PMC9611251; doi:10.3390/pathogens11101204)
Supplement: Supplementary file 1 [file pathogens-11-01204-s001.zip › Table S1. Genbank accession numbers.pdf]

Table S1. GenBank accession numbers of the *Colletotrichum* strains and isolates used for phylogenetic analysis. Sequences generated on this study are highlighted in bold.

Species	Strain	ITS	*gapdh*	*act*	*chs-1*	*tub2*	*his3*	Host	Country	Reference
C. anthrisci	CBS 125334	GU227845	GU228237	GU227943	GU228335	GU228139	GU228041	*Anthriscus sylvestris*	Netherlands	Damm *et al*. (2009)
C. anthrisci	CBS 125335	GU227846	GU228238	GU227944	GU228336	GU228140	GU228042	*Anthriscus sylvestris*	Netherlands	Damm *et al*. (2009)
C. anthrisci	**NAL52**	**MN203633**	**MN207466**	**OM055666**	**OM037442**	**MN207160**	**OM055669**	*Persea americana* cv. Hass	Chile	**This study**
C. anthrisci	**NAL53**	**MN203634**	**MN207467**	**OM055667**	**OM037443**	**MN207161**	**OM055670**	*Persea americana* cv. Hass	Chile	**This study**
C. anthrisci	**NAL54**	**MN203635**	**MN207468**	**OM055668**	**OM037444**	**MN207162**	**OM055671**	*Persea americana* cv. Hass	Chile	**This study**
C. circinans	CBS 221.81	GU227855	GU228247	GU227953	GU228345	GU228149	GU228051	*Allium cepa*	Serbia	Damm *et al*. (2009)
C. dematium	CBS 125.25	GU227819	GU228211	GU227917	GU228309	GU228113	GU228015	*Eryngium campestre*	France	Damm *et al*. (2009)
C. eryngiicola	MFLU 16-1477	KY792726	KY792723	KY792717	KY792720	KY792729	-	*Eryngium* sp.	Russia	Buyck *et al*. (2017)
C. fructi	CBS 346.37	GU227844	GU228236	GU227942	GU228334	GU228138	GU228040	*Malus sylvestris*	USA	Damm *et al*. (2009)
C. gloeosporioides	CBS 112999	JQ005152	JQ005239	JQ005500	JQ005326	JQ005587	JQ005413	*Citrus sinensis*	Italy	Damm *et al*. (2012)
C. hemerocallidis	CBS 130642	JQ400005	JQ400012	JQ399991	JQ399998	JQ400019	-	*Hemerocallis fulva* cv. Kwanso	China	Yang *et al*. (2012)
C. insertae	MFLU 15-1895	KX618686	KX618684	KX618682	KX618683	KX618685	-	*Parthenocissus inserta*	Russia	Hyde *et al*. (2016)
C. jinshuiense	CGMCC 3.18903	MG748077	MG747995	MG747767	MG747913	MG748157	-	*Pyrus pyrifolia* cv. Jinshui	China	Fu *et al*. (2019)
C. kakiivorum	KCTC 46679	LC324781	LC324787	LC324785	LC324783	LC324791	LC324789	*Diospyros kaki*	Korea	Lee and Jung (2018)
C. karsti	CBS 127597	JQ005204	JQ005291	JQ005552	JQ005378	JQ005638	JQ005465	*Diospyros australis*	Australia	Damm *et al*. (2012)
C. lineola	CBS 125337	GU227829	GU228221	GU227927	GU228319	GU228123	GU228025	Apiaceae	Czech Republic	Damm *et al*. (2009)
C. menispermi	MFLUCC 14-0625	KU242357	KU242356	KU242353	KU242355	KU242354	-	*Menispermum dauricum*	Russia	Li *et al*. (2016)
C. orchidis	MFLUCC 17-1302	MK502144	MK496857	MK496853	MK496855	MK496859	-	*Orchis* sp.	Italy	Hyde *et al*. (2020)
C. parthenossicicola	MFLUCC 17-1098	MK629452	MK639362	MK639358	MK639356	MK639360	-	*Parthenocissus quinquefolia*	Russia	Jayawardena *et al*. (2020)
C. quinquefoliae	MFLU 14-0626	KU236391	KU236390	KU236389	-	KU236392	-	*Parthenocissus quinquefolia*	Russia	Li *et al*. (2016)
C. sambucicola	MFLU 16-2675	KY595193	KY595192	KY595190	KY595191	KY595194	-	*Sambucus ebulus*	Italy	Tibpromma *et al*. (2017)
C. sambucicola	MFLUCC 16-1388	KY098781	KY098780	KY098778	KY098779	KY098782	-	*Sambucus ebulus*	Italy	Tibpromma *et al*. (2017)
C. sedi	MFLUCC 14-1002	KM974758	KM974755	KM974756	KM974754	KM974757	-	*Sedum* sp.	Russia	Liu *et al*. (2015)
C. sonchicola	MFLUCC 17-1299	KY962757	KY962754	KY962748	KY962751	-	-	*Sonchus* sp.	Italy	Jayawardena *et al*. (2017)
C. spinaciae	CBS 128.57	GU227847	GU228239	GU227945	GU228337	GU228141	GU228043	*Spinacia oleracea*	Netherlands	Damm *et al*. (2009)
C. zhejiangense	LC13887	MZ595912	MZ664124	MZ664210	MZ799342	MZ674030	MZ673932	Dead leaves of unidentified tree	China	Liu *et al*. (2022)

References

Buyck, B.; Duhem, B.; Das, K.; Jayawardena, R.S.; Niveiro, N.; Pereira, O.L.; Prasher, I.B.; Adhikari, S.; Albertó, E.O.; Bulgakov, T.S.; et al. Fungal biodiversity profiles 21-30. *Cryptogam. Mycol.* **2017**, *38*, 101–146. https://doi.org/10.7872/crym/v38.iss1.2017.101

Damm, U.; Woudenberg, J.H.C.; Cannon, P.F.; Crous, P.W. *Colletotrichum* species with curved conidia from herbaceous hosts. *Fungal Divers.* **2009**, *39*, 45–87.

Damm, U.; Cannon, P.F.; Woudenberg, J.H.C.; Johnston, P.R.; Weir, B.S.; Tan, Y.P.; Shivas, R.G.; Crous, P.W. The *Colletotrichum boninense* species complex. *Stud. Mycol.* **2012**, *73*, 1–36. https://doi.org/10.3114/sim0002

Fu, M.; Crous, P.W.; Bai, Q.; Zhang, P.F.; Xiang, J.; Guo, Y.S.; Zhao, F.F.; Yang, M.M.; Hong, N.; Xu, W.X.; et al. *Colletotrichum* species associated with anthracnose of *Pyrus* spp. in China. *Persoonia* **2019**, *42*, 1–35. https://doi.org/10.3767/persoonia.2019.42.01

Hyde, K.d.; Hongsanan, S.; Jeewon, R.; Bhat, D.J.; McKenzie, E.H.C.; Jones, E.B.G.; Phookamsak, R.; Ariyawansa, H.A.; Boonmee, S.; Zhao, Q. Fungal diversity notes 367–490: taxonomic and phylogenetic contributions to fungal taxa. *Fungal Divers.* **2016**, *80*, 1–270. https://doi.org/10.1007/s13225-016-0373-x

Hyde, K.D.; Norphanphoun, C.; Maharachchikumbura, S.S.N.; Bhat, D.J.; Jones, E.B.G.; Bundhun, D.; Chen, Y.J.; Bao, D.F.; Boonmee, S.; Calabon, M.S.; et al. Refined families of Sordariomycetes. *Mycosphere* **2020**, *11*, 305–1059. https://doi.org/10.5943/mycosphere/11/1/7

Jayawardena, R.S.; Camporesi, E.; Elgorban, A.M.; Bahkali, A.H.; Yan, J.; Hyde, K.D. A new species of *Colletotrichum* from *Sonchus* sp. in Italy. *Phytotaxa* **2017**, *314*, 55–63. https://doi.org/10.11646/phytotaxa.314.1.3

Jayawardena, R.S.; Hyde, K.D.; Chen, Y.J.; Papp, V.; Palla, B.; Papp, D.; Bhunjun, C.S.; Hurdeal, V.G.; Senwanna, C., Manawasinghe, I.S.; et al. One stop shop IV: taxonomic update with molecular phylogeny for important phytopathogenic genera: 76–100 (2020). *Fungal Divers.* **2020**, *103*, 87–218. https://doi.org/10.1007/s13225-020-00460-8

Lee, S.Y.; Jung, H.Y. *Colletotrichum kakivorum* sp. nov., a new leaf spot pathogen of persimmon in Korea. *Mycol. Prog.* **2018**, *17*, 1113–1121. https://doi.org/10.1007/s11557-018-1424-3

Li, G.J.; Hyde, K.D.; Zhao, R.L.; Hongsanan, S.; Abdel-Aziz, F.A.; Abdel-Wahab, M.A.; Alvarado, P.; Alves-Silva, G.; Ammirati, J.F.; Ariyawansa, H.A.; et al. Fungal diversity notes 253–366: taxonomic and phylogenetic contributions to fungal taxa. *Fungal Divers.* **2016**, *78*, 1–237. https://doi.org/10.1007/s13225-016-0366-9

Liu, J.K.; Hyde, K.D.; Jones, E.B.G.; Ariyawansa, H.A.; Bhat, D.J.; Boonmee, S., Maharachchikumbura, S.S.N.; McKenzie, E.H.C.; Phookamsak, R.; Phukhamsakda, C.; et al. Fungal diversity notes 1–110: taxonomic and phylogenetic contributions to fungal species. *Fungal Divers.* **2015**, *72*, 1–197. https://doi.org/10.1007/s13225-015-0324-y

Liu, F.; Ma, Z.Y.; Hou, L.W.; Diao, Y.Z.; Wu, W.P.; Damm, U.; Song, S.; Cai, L. Updating species diversity of *Colletotrichum*, with a phylogenomic overview. *Stud. Mycol.* **2022**, *101*, 1–86. https://doi.org/10.3114/sim.2022.101.01

Tibpromma, S.; Hyde, K.D.; Jeewon, R.; Maharachchikumbura, S.S.N.; Liu, J.K.; Bhat, D.J.; Gareth Jones, E.B.; McKenzie, E.H.C.; Camporesi, E.; Bulgakov, T.S.; et al. Fungal diversity notes 491–602: taxonomic and phylogenetic contributions to fungal taxa. *Fungal Divers.* **2017**, *83*, 1–261. https://doi.org/10.1007/s13225-017-0378-0

Yang, Y.; Liu, Z.; Cai, L.; Hyde, K.D. New species and notes of *Colletotrichum* on daylilies (*Hemerocallis* spp.). *Trop. Plant Pathol.* **2012**, *37*, 165–174. https://doi.org/10.1590/S1982-56762012000300001
